# Fatal non-accidental injury in South Africa: A Gauteng hospital’s perspective on the incidence and fracture types in post-mortem skeletal surveys

**DOI:** 10.4102/sajr.v26i1.2311

**Published:** 2022-02-22

**Authors:** Robyn M. Wessels, Halvani Moodley

**Affiliations:** 1 Department of Diagnostic Radiology, Faculty of Radiation Sciences, University of the Witwatersrand, Johannesburg, South Africa; 2 Charlotte Maxeke Johannesburg Academic Hospital, Johannesburg, South Africa

**Keywords:** fatal non-accidental injury, post-mortem skeletal surveys, PMSS, NAI, live skeletal surveys, LSS

## Abstract

**Background:**

In its severest form, non-accidental injury (NAI) in children is fatal. South Africa has been reported to have double the global average of child homicides. Autopsy is the main investigation in fatal NAI with post-mortem skeletal surveys (PMSS) playing an adjunctive role. Whilst fracture patterns associated with NAI in living patients have been established, this has not been investigated in PMSS in South Africa.

**Objectives:**

To determine the incidence and characteristics of fractures in suspected fatal NAI cases. To calculate the incidence of fractures according to high-, moderate- and low-specificity fracture locations for NAI.

**Methods:**

A retrospective review of all PMSS performed between 01 January 2012 and 03 December 2018 was conducted at the Charlotte Maxeke Johannesburg Academic Hospital.

**Results:**

Of the 73 PMSS, 33 (45.2%) demonstrated fractures. No statistical significance in sex was found: 38 (52.1%) were male and 35 (47.9%) were female (*p* > 0.05). The mean age of those who sustained fractures was 28 months (standard deviation [s.d.]: 21 months). A total of 115 fractures were sustained, of that the top five bones fractured were the ribs 37 (32.2%), parietal bone 13 (11.3%), ulna 13 (11.3%), femur 13 (11.3%), and radius 11 (9.6%). High-specificity fracture locations accounted for 40/133 (30.1%).

**Conclusion:**

The fracture types in PMSS were similar to those in live skeletal surveys. Our study’s fracture rate was higher in comparison to international studies. The PMSS is a valuable adjunct to autopsy in detecting occult fractures of the limbs. We recommend that PMSS be performed in suspected fatal NAI cases at least in children up to 24 months of age.

## Introduction

Child abuse or non-accidental injury (NAI) in its severest form is fatal.^1^ The World Health Organization (WHO) defines child abuse and child maltreatment as:

[*A*]ll forms of physical and/or emotional ill-treatment, sexual abuse, neglect or negligent treatment or commercial or other exploitation, resulting in actual or potential harm to the child’s health, survival, development or dignity in the context of a relationship of responsibility, trust or power.^2^

South Africa (SA) has been reported to have double the global average of child homicides.^3^ Mathews et al. concluded that the rate of child homicide was 5.5 per 100 000 in their study population from two South African provinces in 2009.^3^ The homicide rate was higher in children under the age of five years.^3^ The under five year age group has also been identified in various studies as the prime age group for NAI.^4,5,6,7^ The rate of death as a result of child abuse and neglect was also highest in this age group with nearly half of all child homicide cases (44.5%) being attributed to child abuse or neglect.^3,8^

Autopsy remains the main investigation in cases of suspicious child deaths with post-mortem skeletal surveys (PMSS) playing an adjunctive role.^9,10^ Skeletal surveys are the primary imaging modality to detect occult skeletal injuries in cases of suspected NAI in living and deceased patients.^11^ The use of post-mortem CT and MRI internationally is gaining popularity, whilst locally the use of radiographic PMSS is currently the standard. Some studies have debated the need for routine PMSS, whilst others have highlighted their importance in detecting occult fractures.^12,13^ In SA, however, there is limited capacity for the performance of PMSS as it is a resource constrained environment in terms of the availability of imaging equipment, radiographers and radiologists with experience in post-mortem imaging. Post-mortem CT as the primary post-mortem imaging modality has shown promise in some international centres;^14,15^ however, this is not currently the standard in SA.

The standard views required in PMSS are debated in the literature and varies depending on the individual country or institutional regulations.^12,16,17,18^ In SA, the South African Society of Paediatric Imaging (SASPI) stipulates guidelines for live skeletal surveys (LSS). In Charlotte Maxeke Johannesburg Academic Hospital, modified skeletal survey guidelines from the Red Cross War Memorial Children’s Hospital (RCWMCH) in Cape Town, which are similar to the SASPI guidelines, are used for both living and deceased patients (see Table 1).

**TABLE 1:** Skeletal survey views for non-accidental injury.

Region	Views	Notes
**Axial skeleton**		
Skull†	AP and lateral	
Chest	AP, bilateral oblique ribs	
Abdomen	AP	To demonstrate spine, pelvis, hips and lower ribs
Spine	Lateral	
**Appendicular skeleton**		
Upper and lower limbs	AP	Centre on joints
Hands and feet	AP	
Knees, ankles, wrists and elbows	Lateral‡	

*Source*: Modified from RCWMCH guidelines

AP, anteroposterior.

† , In LSS, a CT brain is performed, however we have modified this protocol for PMSS.

‡ , These views are added at the discretion of the attending radiologist.

The procedure of performing a PMSS is more cumbersome and time-consuming than performing a LSS. The post-mortem condition of the patient (rigor mortis, decomposition, post autopsy changes) provides unique challenges in obtaining and maintaining the diagnostic quality of the PMSS images. Hughes-Roberts et al. found a larger proportion of significant artefacts in the PMSS when compared to the LSS.^9^ Despite these factors, PMSS remains the current standard for occult fracture detection and fracture dating in suspected fatal cases of NAI in SA.^19^

The patterns of fractures associated with NAI in living patients have been published internationally.^17,18,20^ These have been divided into high-, moderate- and low-specificity groups for NAI as follows: high-specificity fractures include classic metaphyseal lesions, rib fractures (especially posterior-medial), scapular fractures, spinous process fractures and sternal fractures.^17,18,20^ Moderate-specificity fractures include multiple fractures (especially bilateral), fractures of different ages, vertebral body fractures and subluxations, digital fractures and complex skull fractures.^17,18,20^ Low-specificity fractures comprise subperiosteal new bone formation, clavicular fractures, long bone shaft fractures and linear skull fractures.^17,18,20^ In contradistinction, only a single South African study was published in 2007 detailing the fracture patterns sustained in living patients at the RCWMCH.^6^ The fracture patterns in PMSS in SA have not been investigated to date.

The paucity of research on the fractures sustained in fatal NAI and the debate regarding the validity of the PMSS provided the motivation for our study framework in a South African tertiary hospital setting.

The aim and objectives of this descriptive study were therefore to determine the incidence and characteristics of the fractures in child fatalities suspected of having been subjected to NAI, and to establish how many fractures occurred in high-, moderate- and low-specificity locations for NAI, as per O’Connor et al.^21^ The degree of concordance between two readers of varying reporting experience levels was also assessed.

## Materials and methods

A retrospective review of all the consecutive PMSS referred to our hospital by the local forensic pathology services performed over a six-year period (01 January 2012 to 03 December 2018) was conducted. The PMSS of all child fatalities with suspected NAI, those who were found to be deceased in suspicious or unknown circumstances or those who had died unexpectedly, were included in the study. The study included all children from the age of birth to adolescence. The WHO definition of adolescence, those aged between 10 and 19 years, was employed. Where the date of birth was not available, estimation of age was made using a bone age atlas.^22^

All cases on film (prior to April 2016) and those saved digitally onto the hospital’s picture archiving and communication system (PACS) (after 01 April 2016) were assessed. The views were acquired according to the protocol detailed in Table 1.

Each skeletal survey was read independently by a radiology registrar (year three of study) and a paediatric radiologist (four years of experience) blinded to all clinical details except sex and age. A standardised checklist for reporting was used by the readers for data collection, which assessed demographic details, technical aspects and skeletal survey findings including fracture dating (i.e., acute, healing and healed fractures) according to Paddock et al.^17^

Statistical analysis was performed with STATA 15. The descriptive statistics: mean, median, standard deviation (s.d.) and interquartile range (IQR) were used to describe the continuous variables including age and the number of fractures per patient. Frequencies and proportions were used to describe the type of fractures and other categorical variables. Incidence rates were calculated for the number of fractures in the study population, first overall as well as by the type of fracture. Associations between categorical variables were tested using the Fisher’s exact test. Cohen’s Kappa was used to determine the concordance between readers. Tests were evaluated at a 5% level of significance.

### Ethical considerations

Ethical approval was granted by the Human Research Ethics Committee (Medical) of the University of the Witwatersrand. Clearance number: M181112. All data were anonymised.

## Results

### Technical aspects

A total of 73 PMSS were analysed. With regards to the technical aspects assessed: 21/73 surveys (28.8%) had adequate views, 52/73 (71.2%) had inadequate views, of which 39/52 (75%) were due to incorrect positioning. Artefacts were found in 54/73 (74%) of the PMSS. The majority of the artefacts encountered were on account of identification bands 34/54 (63%) and medical tubes and lines 11/54 (20.4%).

### Demographics

No statistical significance in sex was found: 38/73 (52.1%) were male and 35/73 (47.9%) were female (*p* > 0.05). The mean age of the study population was 26 months (s.d.: 27 months). The age range was 0 months – 13 years. The median age of the study population was 14 months. The median age of those who sustained fractures was 24 months. A total of 39 (53.4%) cases were under the age of 24 months.

### Fracture characteristics

**FIGURE 1:**
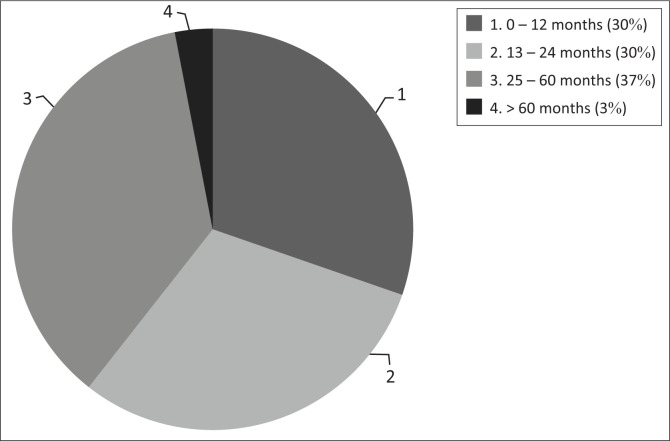
Cases with fractures according to age group (*N* = 33/73).

A total of 73 PMSS yielded fractures in 33 cases (45.2%). Overall, 115 fractures were sustained. The mean age of those who sustained at least one fracture was 28 months (s.d.: 21 months). A comparison between the cases who sustained fractures according to the different age groups is illustrated in Figure 1. There was no statistical significance when comparing the ages of those who sustained fractures to those who did not (*p* > 0.05).

When considering the sex and age comparatively, the mean age of those with at least one fracture in girls was 27 months (s.d.: 23 months) and in boys was 30 months (s.d.: 19 months). The mean number of fractures sustained per case was 3.5 (115/33). The mean number of fractures sustained when comparing girls to boys were as follows: boys 2.7 (s.d.: 4.3) and girls 1.7 (s.d.: 2.5).

The most common fracture locations per region were: the chest 39/115 (33.9%), skull 20/115 (17.4%), upper limbs 31/115 (27%) and lower limbs 25/115 (21.7%) (see Table 2).

**TABLE 2:** Fractures according to region and site.

Region	Site	Number	%
Skull	Frontal bone	4	3.5
	Parietal bone	13	11.3
	Occipital bone	3	2.6
Chest	Ribs	37	32.2
	Clavicle	1	0.9
	Scapula	1	0.9
Upper limbs	Humerus	6	5.2
	Radius	11	9.6
	Ulna	13	11.3
	Metacarpals	1	0.9
Lower limbs	Femur	13	11.3
	Tibia	8	7.0
	Fibula	2	1.7
	Metatarsals	2	1.7

**Total**	**-**	**115**	**-**

The top five most common fractured bones were: the ribs 37/115 (32.2%), parietal bone 13/115 (11.3%), ulna 13/115 (11.3%), femur 13/115 (11.3%) and radius 11/115 (9.6%) (see Table 2). There was no statistical significance in the numbers of the fractures between the top five most common fractured bones (*p* > 0.05).

**FIGURE 2:**
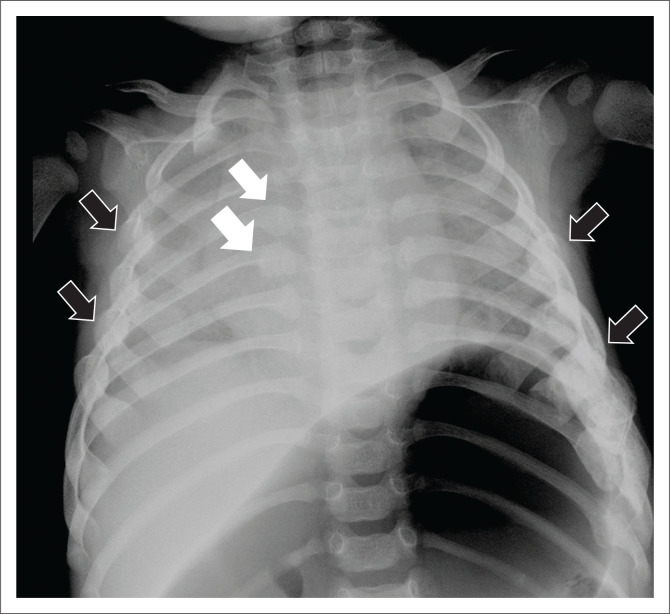
Three-year-old female with bilateral anterior (black arrows) and posterior healed rib fractures (white arrows) on an AP chest view. These are high-specificity fractures for NAI.

**FIGURE 3:**
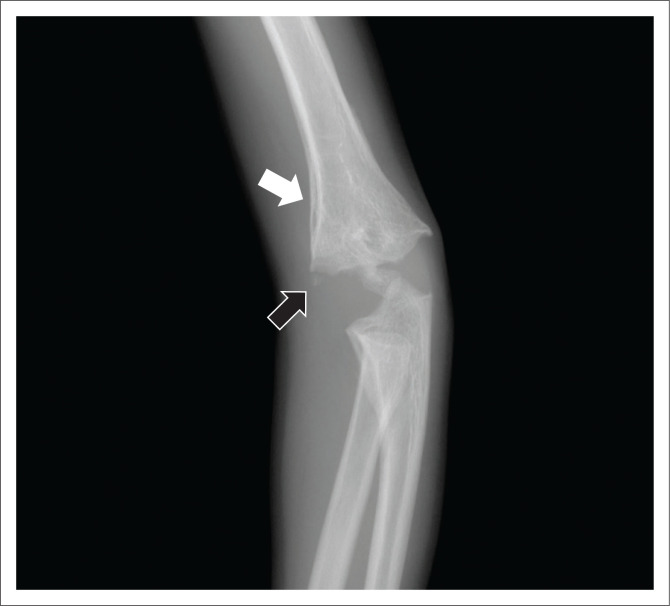
Three-year-old male with a medial distal humerus metaphyseal corner fracture (black arrow) and a periosteal reaction medially (white arrow) on an AP view of the left humerus. The metaphyseal corner fracture is a high-specificity fracture location for non-accidental injury (NAI).

**FIGURE 4:**
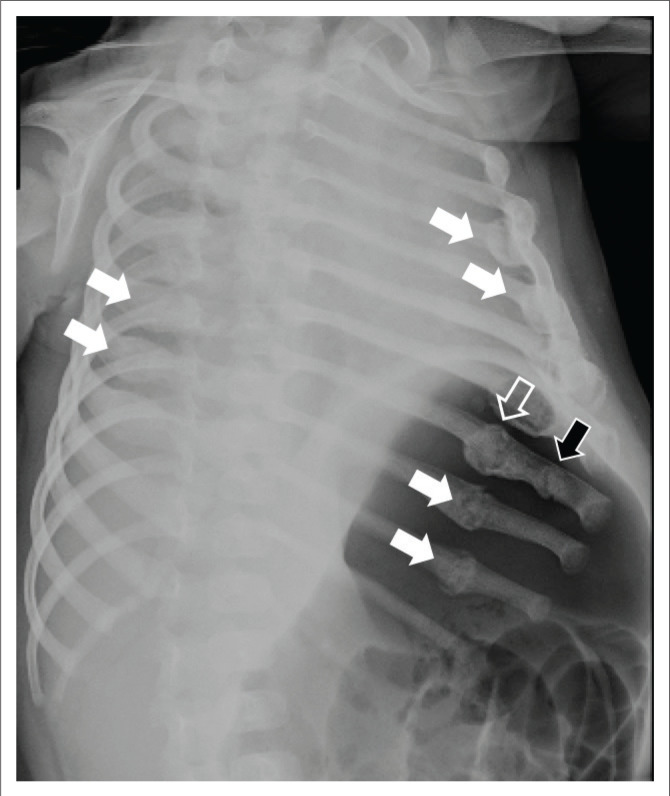
Left oblique rib view of a 3-year-old girl with multiple rib fractures (white arrows) in various stages of healing. There is a more acute fracture of the left eighth rib with minimal callus formation (black arrow) and a chronic healing second fracture in this rib with more advanced callus formation (open arrow).

**FIGURE 5:**
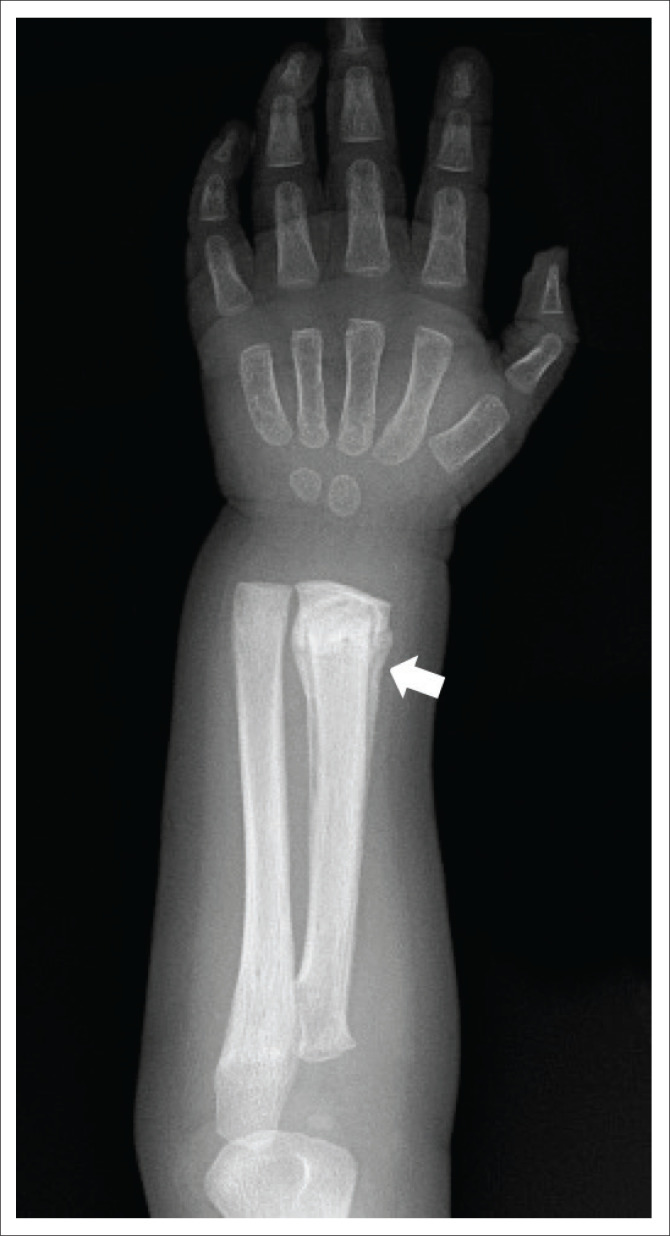
Five-year-old-male with a distal radial diaphyseal fracture with callus formation (white arrow) indicating a healing fracture on an AP view of the right forearm. This is a low-specificity fracture for non-accidental injury.

**FIGURE 6:**
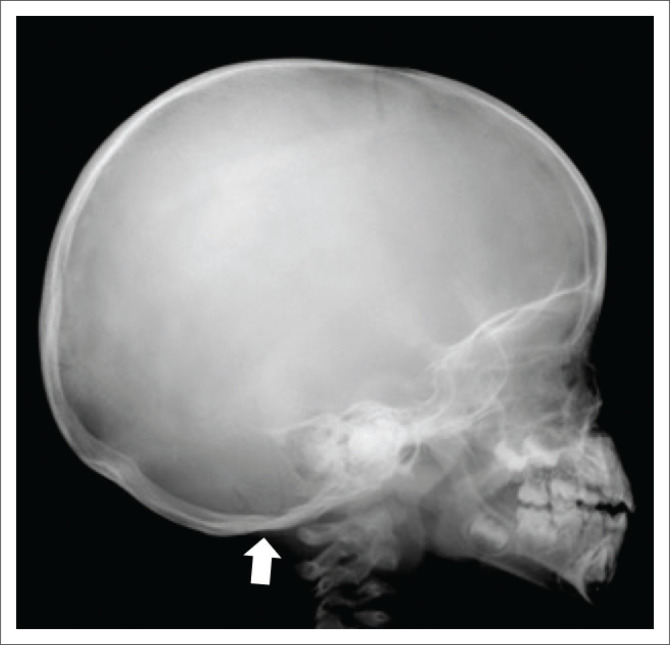
Two-year-old female with a linear occipital bone fracture (white arrow) on a lateral skull view. This is a low-specificity fracture for non-accidental injury.

High-specificity fractures accounted for 40/133 (30.1%) of the fracture locations (Figures 2 and 3), moderate-specificity fractures accounted for 30/133 (22.6%) and low-specificity fractures accounted for 63/133 (47.4%) of fracture locations (see Table 3; Figures 4, 5 & 6).

**TABLE 3:** Specificity of fracture locations and reader agreement.

Specificity	Fracture/Location/Type	Number	%
High	Classic metaphyseal lesions	2	1.5
	Rib fractures (especially posteromedial)	37	27.8
	Scapular fractures	1	0.8
	Spinous process fractures	0	0.0
	Sternal fractures	0	0.0
Total		40	-
Moderate	Multiple fractures (especially bilateral)	21	15.8
	Fractures of different ages	5	3.8
	Vertebral body fractures and subluxations	0	0.0
	Digital fractures	0	0.0
	Complex skull fractures	4	3.0
Total		30	-
Low	Subperiosteal new bone formation	25	18.8
	Clavicular fractures	1	0.8
	Long bone shaft fractures	26	19.5
	Linear skull fractures	11	8.3
Total		63	-

**Grand total**		**133**	**-**

*Source:* Adapted from O’Connor J CJDfIKP, ed. Diagnostic imaing of child abuse. St Louis, MO: Mosby; 1998, p. 168–177.

Note: Reader agreement (high): Kappa value – 0.73; Reader agreement (moderate): Kappa value – 0.9; Reader agreement (low): Kappa value – 0.8.

### Fracture dating

With regards to fracture ages, 17/33 (51.5%) cases demonstrated acute fractures, 16/33 (48.5%) healing fractures and 5/33 (15.2%) chronic fractures (see Figure 4). Acute fractures were found in the ribs (5), clavicle (1), scapula (1), humerus (1), radius (4), ulna (3), femur (4), tibia (4) and fibula (2). Healing fractures were found in the ribs (6), humerus (3), ulna (5), radius (4), metacarpal (1), femur (4), tibia (2) and metatarsals (2). Chronic fractures were found in the ribs (1), humerus (2), radius (1), ulna (2) and femur (1).

### Reader agreement

When comparing agreement on the incidence of the top five bones fractured, the reader agreement (Kappa values) was as follows: ribs 0.7, parietal bone 0.87, ulna 0.85, femur 0.87 and radius 0.93.

The mean reader agreement (Kappa values) for high-, moderate- and low-specificity fracture locations for NAI was 0.73, 0.9 and 0.8, respectively (see Table 3).

## Discussion

This study is the first to document the fracture types and characteristics in deceased children being investigated for suspected fatal NAI in a South African population.

The assessment of the adequacy of the PMSS identified a high number with inadequate views, three-quarters of which were due to poor positioning. This is not an unusual finding in post-mortem imaging, as an audit of skeletal surveys in the United Kingdom (UK) three years after the British Society of Paediatric Radiology (BPSR) standards were published, revealed that only 15% included all views.^23^ This reiterates the widespread challenges faced and the need for quality assurance when performing PMSS.^9,23^ As these studies were conducted over a number of years with various radiographers and radiologists in attendance, all of whom had varying levels of training, this may have accounted for the higher incidence of inadequate positioning. Currently in our department, PMSS and LSS are only performed during working hours by radiographers with the most experience in this type of imaging, where possible.

Each skeletal survey is approved by a consultant radiologist. Because of the small number of paediatric radiologists in SA, and in our hospital specifically, not every skeletal survey can be reviewed by this type of specialist. It is imperative that there is adherence to, and consistency of the imaging generated according to the local protocol. This will facilitate obtaining PMSS of sufficient diagnostic quality to aid accurate interpretation by radiologists with varying levels of experience.^23,24,25^ Additionally, a significant number of artefacts were recorded; however, these are similar to that reported by Hughes et al.^9^

The study findings were compared specifically to two key studies as detailed in Table 4. We found no differences between the age or sex of those who sustained fractures and those without fractures. The mean age of our study population was younger compared to the van As et al. study; however, much younger children were reported by Hughes et al.^6,9^ We have established that there is a much higher yield for fractures on PMSS, almost four times than on LSS in the Cape Town population and five times more than on PMSS in the UK.^6,9^ This initial finding should be corroborated with future comparative studies on LSS and PMSS in SA. Despite the differences in these two countries findings, we concur that fatal NAI is more common in younger children and the injuries sustained tend to be more severe.^6,9^

**TABLE 4:** Comparison between this study’s main findings and key studies.

Author, (year), region	Number of cases		Live skeletal survey results (LSS)	Post-mortem skeletal survey results (PMSS)	Fracture characteristics (Anatomical regions)
	*n*	mean age ± s.d. (months)			
The current study, (2022), Johannesburg, SA	73	28 ± 21	N/A	73 surveys	21 (21.8%) with multiple fractures
				33 (45.2%) abnormal surveys	37/115 (32.2%) rib fractures
					20/115 (17.4%) skull fractures
					31/115 (27%) upper limb long bone fractures
					25/115 (21.7%) lower limb long bone fractures
Hughes-Roberts, (2012), Cambridge, UK	195	7.1 ± 9.2	67 surveys	128 surveys	LSS:
			16 (23.8%) abnormal surveys	11 (8.6%) abnormal surveys	4 multiple fractures
					6 skull fractures
					9 long bone fractures
					PMSS:
					5 multiple fractures
					5 skull fractures
					4 long bones fractures
Van As, (2007), Cape Town, SA	1037	44.8 ± 49.2	121 surveys	N/A	21 (17.3%) with multiple fractures
			(11.7%)		57/149 (38.2%) skull fractures
			sustained fractures		29/149 (19.4%) upper limb long bone fractures
					26/149 (17.4%) lower limb long bone fractures

The characteristics of fractures in our study were concordant with the literature in that the most common regions fractured were the chest, the skull and the limbs.^4,6,7,20,26^ Most articles were based on findings in LSS with a few on PMSS. We demonstrated similar findings to those of Van As et al. in that some high-specificity fracture locations for NAI (metaphyseal corner fractures, scapula and sternal fractures) were rare in this series (see Table 3), however high-specificity fracture locations overall accounted for just over a third of the injuries.Only rib fractures were more common in our series compared to Van As et al., whereas skull fractures were in the majority in the latter. This may show a possible difference in fracture patterns sustained per province in SA. The need for future local studies to establish the spectrum of fracture patterns in our population at multiple centres is emphasised.

In some studies, the need for routine PMSS has been debated.^12^ McGraw et al. concluded that the addition of a PMSS added valuable additional information to the autopsy findings in cases of suspected fatal NAI. In his study about post-mortem radiography, 92% of the extremity fractures identified were metaphyseal corner fractures.^13^ These types of fractures are highly specific for NAI and are often difficult to detect on routine autopsy.^10,13^ In this study, however we found fewer metaphyseal corner fractures compared to the studies performed by McGraw et al. and Kleinman et al.^13,27^ More importantly, we found that overall there was a high incidence of the limb fractures in our PMSS series. Many of the limb fractures would have otherwise been occult at autopsy, since these regions are not routinely dissected. This and the further findings of multiple fractures, fractures at varying stages of healing and specifically the high yield of high-specificity fracture locations on PMSS in our cohort illustrates the utility of PMSS, as a necessary adjunct in the evaluation of fatal NAI in identifying and documenting the extent of injuries for the ensuing medico-legal proceedings.

We assessed fracture dating which has not been investigated in our population previously. Acute fractures made up over half of those who sustained fractures. This possibly indicates the severity of the injuries sustained and that a proportion of patients do not survive to recover from them. The overall number of fractures in multiple stages of healing could allude to the repeated abuse in these cases. Fracture dating in PMSS remains an important observation to make in suspected cases of NAI, as determining the chronicity of fractures contributes to the medico-legal case by providing evidence of previously sustained injury.^17,18^

It is important to report on reader agreement to indicate if the assessment criteria are robust and valid in clinical practice. This has not been assessed in our setting before. This study showed substantial to almost perfect agreement between the readers (see Table 3). The substantial agreement was found in the identification of high-specificity fractures. This could be because of the finite details being overlooked by less experienced readers. This finding speaks to the challenges of offering these much-needed services in a country with a higher prevalence of NAI compared to worldwide, with limited paediatric radiology expertise in PMSS. The need for dissemination of this training and expertise to general radiologists and radiology registrars should therefore be encouraged.

## Limitations

The most notable limitation to this study is its retrospective nature which may have introduced bias. The study was conducted at a single centre. Furthermore, no comparison of PMSS to LSS, autopsy findings or the final medico-legal investigation outcome with confirmed cases of fatal child abuse was performed.

## Conclusion and recommendations

This study analysed the first and largest number of PMSS in SA to date. In comparison to international studies, the study population’s fracture rate was notably higher. We have shown that in cases of suspected NAI, the types of fractures found in PMSS were similar to those seen in LSS, and predominantly occurred in those under 24 months of age. Whereas some of the classically described high-specificity fracture locations for NAI in the international literature, were rare. Future local studies are needed to investigate possible differences in the types of fractures sustained according to region.

Post-mortem skeletal surveys in the South African setting is a valuable adjunct to autopsy in detecting occult fractures of the limbs, which formed a large portion of the fractures that would not have been discovered or routinely dissected at autopsy. We therefore recommend PMSS at least be performed in suspected fatal NAI cases in children up to 24 months of age.

Standardised radiographic technique as per the local guidelines and quality assurance of PMSS is vital to obviate factors that affect interpretation. Furthermore, in our resource limited setting, the encouragement and continuous education and training of radiographers regarding imaging technique, general radiologists and radiology registrars on interpreting PMSS remain key to improving the investigation of these cases.
